# Unraveling the Underlying Mechanisms of Loneliness, Social Isolation, and Cognitive Decline: A Systematic Review

**DOI:** 10.1093/geroni/igaf122.905

**Published:** 2025-12-31

**Authors:** Kexin Yu, Karina Van Bogart, Jee Eun Kang, Harry Taylor, Karra Harrington, Lisa Silbert, Ihab Hajjar, Hiroko Dodge

**Affiliations:** UT Southwestern Medical Center, Dallas, Texas, United States; Northwestern University, Chicago, Illinois, United States; The Pennsylvania State University, University Park, Pennsylvania, United States; University of Toronto, Toronto, Ontario, Canada; Cogstate, Moses Lake, Washington, United States; Oregon Health & Science University, Portland, Oregon, United States; UT Southwestern Medical Center, Dallas, Texas, United States; Massachusetts General Hospital, Harvard Medical School, Boston, Massachusetts, United States

## Abstract

Despite extensive evidence linking loneliness and social isolation to cognitive decline in later life, the underlying pathophysiological and psychosocial mechanisms remain unclear. This systematic review synthesizes existing research to clarify these pathways. A comprehensive search was conducted across PubMed, Web of Science, EMBASE, and PsycINFO until October 2024. Using Covidence for literature management, we included peer-reviewed studies in English involving human participants aged 50+. Studies examining psychosocial pathways required mediation analyses, whereas studies on physiological pathways were included if outcomes were related to dementia pathology. From 4,314 unique articles, 40 met the inclusion criteria. Identified pathophysiological mechanisms included amyloid-beta and tau levels, grey matter atrophies, white matter hyperintensities, dementia-related gene expression, reduced brain-derived neurotrophic factor (BDNF), hypothalamic-pituitary-adrenal (HPA) axis dysregulation, and impaired cerebral blood flow. Psychosocial mediators encompassed depression, anxiety, sleep disturbances, physical inactivity, personality traits, and diminished sense of control. The presentation will contrast the unique pathways for loneliness and social isolation. Evidence suggests loneliness and social isolation are linked to decreased resiliency to neuropathological changes and to behavioral and psychological factors that accelerate cognitive decline. However, existing research relies heavily on self-report measures and does not account for potential confounders like pre-existing health conditions, which limits causal inference. By integrating evidence across disciplines, our findings underscore the multifaceted impact of social disconnection on cognitive health. Future research should prioritize rigorous longitudinal designs, including multi-modal biomarkers, both subjective and objective psychosocial measures, and potential confounders in analyses. Such work would inform targeted interventions that promote cognitive resilience.

